# Contribution of Litter and Root to Soil Nutrients in Different Rocky Desertification Grasslands in a Karst Area

**DOI:** 10.3390/plants13162329

**Published:** 2024-08-21

**Authors:** Yuefeng Wang, Jigao Wang, Yini Wang, Xiaojing Wang, Baocheng Jin, Chao Chen, Xuechun Zhao

**Affiliations:** 1 College of Animal Science, Guizhou University, Guiyang 550025, China; gs.yuefengwang22@gzu.edu.cn (Y.W.); wjg@imde.ac.cn (J.W.); bcjin@gzu.edu.cn (B.J.); chenc@gzu.edu.cn (C.C.); 2 Key Laboratory of Mountain Surface Processes and Ecological Regulation, Institute of Mountain Hazards and Environment, Chinese Academy of Sciences, Chengdu 610041, China; 3 Guizhou Grassland Technology Test and Extension Station, Guiyang 550025, China; 19311970957@163.com; 4 Key Laboratory of Plant Resource Conservation and Germplasm Innovation in Mountainous Region (Ministry of Education), Institute of Agro-Bioengineering, Guizhou University, Guiyang 550025, China; xjwang8@gzu.edu.cn

**Keywords:** rocky desertification grasslands, litter, root, nutrient release

## Abstract

Litter and root decomposition is an important source of soil organic matter and nutrients. To ascertain the contribution of litter and root to natural grassland nutrients in rocky desertification areas, from March 2017 to January 2018, the continuous soil column method, collector method, and litter decomposition method were used to study the soil nutrients, litter and root biomass, decomposition, and nutrient release of potential, moderate, and severe rocky desertification grasslands, as well as their responses to rocky desertification. The results showed that the litter and root decomposition rate showed a trend of being first fast and then slow, and the decomposition rate of litter and root was greater than 50% after 300 days. The annual litter decomposition rates of potential, moderate, and severe rocky desertification grasslands were 69.98%, 62.14%, and 49.79%, respectively, and the annual decomposition rates of root were 73.64%, 67.61%, and 64.09%, respectively. With a deepening degree of rocky desertification, the litter and root decomposition rate decreased. The decomposition coefficients, *k*, of litter in potential, moderate, and severe rocky desertification grasslands were 1.128, 0.896, and 0.668, respectively, and the decomposition coefficients, *k*, of root were 1.152, 1.018, and 0.987, respectively. The nutrient release processes of litter and root were different, and the release mode ultimately manifests as “release”. In rocky desertification grasslands, the organic carbon (OC), total nitrogen (TN), total phosphorus (TP), and total potassium (TK) released by litter and root decomposition were 18.93–263.03 g·m^−2^·yr^−1^, 1.79–5.59 g·m^−2^·yr^−1^, 0.18–0.47 g·m^−2^·yr^−1^, and 0.66–3.70 g·m^−2^·yr^−1^, respectively. The contribution of root to soil nutrients was greater than that of litter. The degree of rocky desertification was negatively correlated with the biomass, decomposition rate, and nutrient return amount of litter and root. The results of this study provide direct field evidence and illustrate the contribution of litter and root decomposition in rocky desertification grasslands to soil nutrients.

## 1. Introduction

Nutrient cycling is a primary function of terrestrial ecosystems. The production and decomposition of litter and root are the primary nutrient-cycling pathways in terrestrial ecosystems [1], and they jointly determine the vegetation turnover and nutrient cycling of an ecosystem [2]. Totals of 31% and 43% of the net primary productivity of plants are allocated to the production of leaf and root, respectively. Leaching, decomposition, and turnover of leaf and root return the nutrients they fix to the soil [3,4]. Among the nutrients absorbed by plants, approximately 90% of N, P, and K, and more than 65% of mineral elements originate from litter nutrient recycling [5]. In addition, nutrients such as carbon and nitrogen returned to the soil from root decomposition and turnover are greater than the inputs from aboveground litter [6,7]. The nutrient turnover process of litter and root is primarily affected by comprehensive factors such as the quality of litter and root, climate, and soil properties (soil physical and chemical properties, microorganisms, and animals, etc.) [8,9].

Karst landforms account for 15.6% of the world’s land area and are widely distributed throughout the world [10]. Guizhou Province contains the most typical karst landform in China, with 2.47 × 10^4^ km^2^ of karst grasslands, accounting for 14.02% of the national land area. Approximately 70% of karst grasslands have varying degrees of rocky desertification [11]. Among them, potential (bare rock rate is 20–30%), mild (bare rock rate is 31–50%), moderate (bare rock rate is 51–70%), and severe (bare rock rate is greater than 70%) rocky desertification grasslands account for 30.77%, 23.78%, 11.37%, and 4.62% of karst grasslands, respectively. Grasslands are the primary vegetation type in karst areas, and they have a huge root biomass and high litter production, which play important roles in nutrient cycling and carbon sequestration. Rocky desertification areas have poor soils fertility and water-holding capacities; hence, the roles of root growth, decomposition, and turnover need to be determined. These processes connect aboveground plants and soils [12], and the substances released through litter decomposition replenish soil nutrients and play important roles in maintaining the productivity of rocky desertification grasslands.

Currently, there are a large number of studies that have been conducted in China and abroad concerning the decomposition and nutrient release of litter and root, and they have primarily focused on the reserves, decomposition, water-holding characteristics, and the stoichiometry of litter and root in forest [13,14], farmland [15,16], and grassland [17,18] ecosystems. However, studies in karst mountainous areas with shallow soil layers and discontinuous vegetation have been rare. Previous studies have shown that roots contribute more to soil nutrients than litter in non-karst areas [6,7], and it is uncertain whether this is the case for grasslands in rocky desertification areas. Therefore, in this study, potential, moderate, and severe rocky desertification natural grasslands are used as the research objects, and the collector method, the continuous soil column method, and the decomposition bag method are used to study the biomass dynamics, decomposition, turnover, and nutrient release of litter and root. This was conducted to analyze the relationship between the degree of rocky desertification and the biomass, annual decomposition rate, and nutrient return amount of litter and root. The aim of this study is to achieve the following: (1) to clarify the yield and biomass dynamics of litter and root; (2) to evaluate the decomposition, turnover, and nutrient release of litter and root in different rocky desertification grasslands; and (3) to determine the differences in the contribution of litter and root to soil nutrients in different rocky desertification grasslands.

## 2. Results

### 2.1. Soil Nutrients

**Figure 1:**
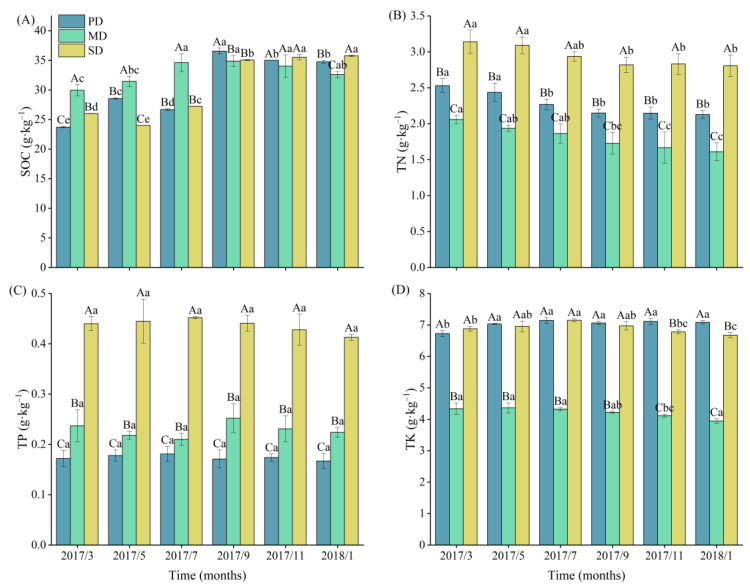
Soil organic carbon (SOC, **A**), total nitrogen (TN, **B**), total phosphorus (TP, **C**), and total potassium (TK, **D**) content in potential (PD), moderate (MD), and severe (SD) rocky desertification grasslands. Different uppercase letters indicate significant differences at a sampling time for different grassland types, while different lowercase letters indicate significant differences at different sampling times for the same grassland type. *x*-axis indicates year/month.

The monthly average values of the SOC in potential, moderate, and severe rocky desertification grasslands were 30.87, 32.92, and 30.60 g·kg^−1^, respectively. The monthly average values of the TN were 2.28, 1.81, and 2.94 g·kg^−1^, respectively. The monthly average values of the TP were 0.17, 0.23, and 0.44 g·kg^−1^, respectively. The monthly average values of the TK were 7.03, 4.22, and 6.90 g·kg^−1^, respectively. The maximum values of the total nitrogen in the soils of the three rocky desertification grasslands all appeared in March, and the minimum values all appeared in January. There were significant differences in the TN and TP in the soil of different levels of rocky desertification grasslands in each month. The TN and TP in the soil of the severe rocky desertification grassland were significantly greater than those in the potential and moderate rocky desertification grasslands (*p* < 0.05) (Figure 1).

### 2.2. Litter and Root Biomass and Dynamics

**Figure 2:**
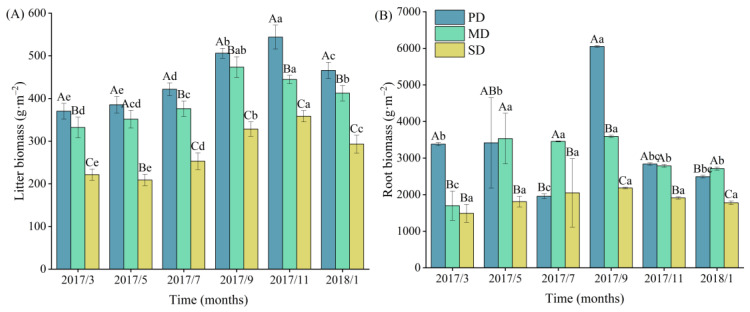
Litter (**A**) and root (**B**) biomass in potential (PD), moderate (MD), and severe (SD) rocky desertification grasslands. Different uppercase letters indicate significant differences at a sampling time for different grassland types, while different lowercase letters indicate significant differences at different sampling times for the same grassland type. *x*-axis indicates year/month.

With the increase in rocky desertification, both litter and root biomass showed decreasing trends. The litter biomass values in the potential, moderate, and severe rocky desertification grasslands were 448, 398, and 277 g·m^−2^, respectively, and the root biomass values were 3356, 2962, and 1869 g·m^−2^, respectively. From March to January of the following year, both litter and root biomasses showed first increasing trends and then decreasing trends. The litter biomass values in the potential, moderate, and severe rocky desertification grasslands reached maximums from September to November with maximum values of 543, 473, and 358 g·m^−2^, respectively. The root biomass values in the potential, moderate, and severe rocky desertification grasslands all reached maximums in September with maximum values of 6051, 3591, and 2181 g·m^−2^, respectively (Figure 2).

### 2.3. Decomposition of Litter and Root

**Figure 3:**
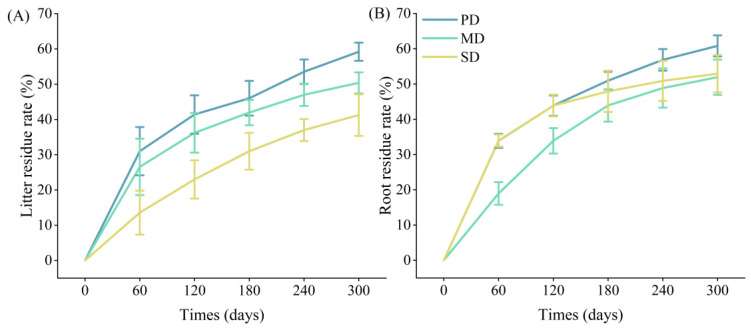
Litter (**A**) and root (**B**) decomposition rates in the potential (PD), moderate (MD), and severe (SD) rocky desertification grasslands.

The decomposition rates of litter and root showed gradually increasing trends with the decomposition time. During the initial stage of decomposition (0–60 days), the decomposition rates of litter and root were relatively fast and gradually slowed down as the decomposition time passed. After 300 days of decomposition, the decomposition rates of litter in the potential, moderate, and severe rocky desertification grasslands were 59.20%, 50.38%, and 41.24%, respectively, and the decomposition rates of roots were 60.83%, 51.93%, and 52.93%, respectively (Figure 3).

Table 1 shows that both the litter and root production were in the order of potential rocky desertification > moderate rocky desertification > severe rocky desertification. The annual decomposition amount of litter gradually decreased with the deepening of rocky desertification, and the root annual decomposition amount first increased and then decreased with the deepening of rocky desertification. The turnover rate of litter was the highest in the severe rocky desertification grasslands (0.57 yr^−1^), which was significantly greater than that in the potential and moderate rocky desertification grasslands (*p* < 0.05). The turnover rate of root was the highest in the potential rocky desertification grasslands (1.51 yr^−1^), and there was no significant difference with the litter turnover rate in the moderate rocky desertification grasslands. However, there was a significant difference with that in the severe rocky desertification grasslands (*p* < 0.05) (Table 1).

The Olson index simulation showed that the decomposition coefficients, *k*, of litter in the potential, moderate, and severe rocky desertification grasslands were 1.128, 0.896, and 0.668, respectively, and the decomposition coefficients of root were 1.152, 1.018, and 0.987, respectively. When 50% of the litter was decomposed, this took 0.89, 1.15, and 1.16 years, respectively, in the potential, moderate, and severe rocky desertification grasslands. When 50% of the root was decomposed, this took 0.49, 0.57, and 0.66 years, respectively, in the potential, moderate, and severe rocky desertification grasslands. The annual litter decomposition rates in the potential, moderate, and severe rocky desertification grasslands were 69.98%, 62.14%, and 49.79%, respectively, and the annual decomposition rates of root were 73.64%, 67.61%, and 64.09%, respectively (Table 2).

### 2.4. Nutrient Release from Litter and Root

**Figure 4:**
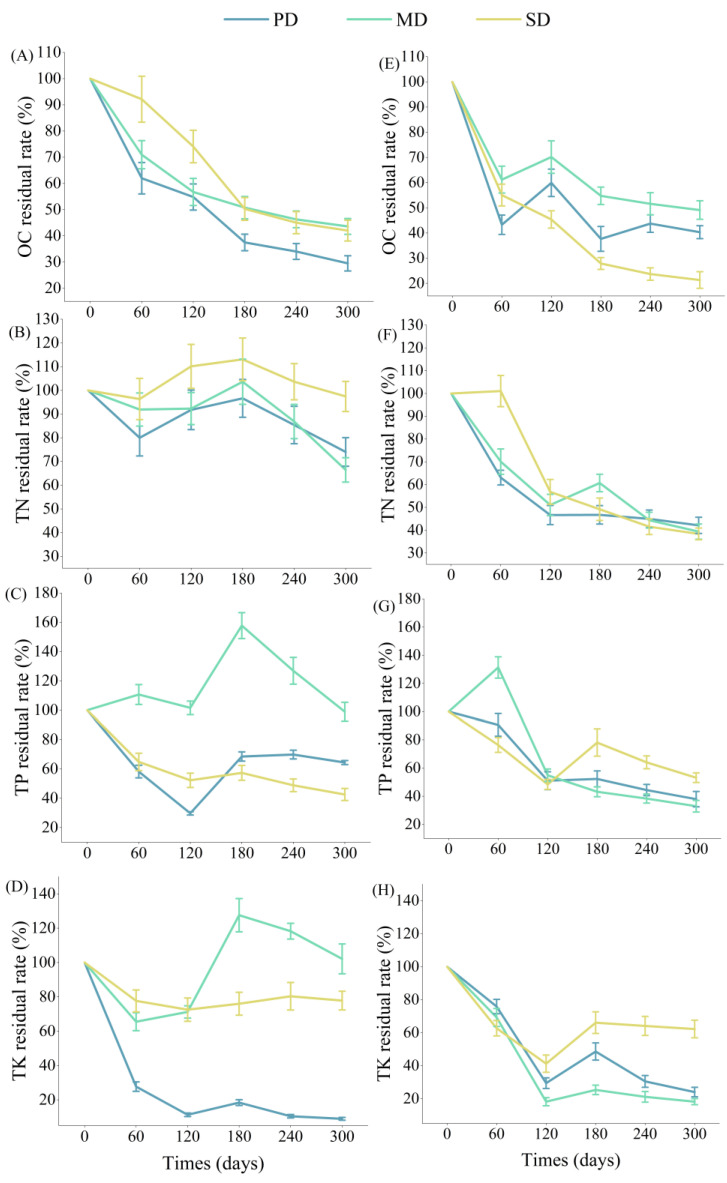
Litter (**A**–**D**) and root (**E**–**H**) organic carbon (OC), total nitrogen (TN), total phosphorus (TP), and total potassium (TK) residue rates in potential rocky desertification (PD), moderate rocky desertification (MD), and severe rocky desertification (SD) grasslands.

The OC, TP, and TK residue rates of the litter decreased significantly in 120 days. The TN, TP, and TK residue rates of the root had the same trend as the litter in 120 days. After 120 days of litter and root decomposition (Figure 4), the OC residual rate of the root in potential and moderate rocky desertification grasslands was greater than the litter; the TN residue rate of the litter in potential, moderate, and severe rocky desertification grasslands was greater than the root; and the TP and TK residual rates of the root in moderate and severe rocky desertification grasslands was greater than the litter.

The litter OC in the potential, moderate, and severe rocky desertification grasslands all showed “leaching-release” dynamics (Figure 4A). The root OC in the potential and moderate rocky desertification grasslands showed “leaching-enrichment-release”, and the severe rocky desertification grasslands showed “leaching-release” (Figure 4E). The annual release rates of litter OC in the three rocky desertification grasslands were 70.49%, 56.40%, and 58.00%. The annual release rates of root OC were 59.68%, 50.89%, and 78.68%.

The litter TN in the potential, moderate, and severe rocky desertification grasslands all showed “leaching-enrichment-release” (Figure 4B). The root TN showed great differences in the root nutrient release process in the potential, moderate, and severe rocky desertification grasslands, showing “leaching-release”, “leaching-enrichment-release”, and “enrichment-release”, respectively. The release rates of the root TN were 57.87%, 60.61%, and 61.64% (Figure 4F).

TP showed “leaching-enrichment-release” during the nutrient release process of litter and root in potential and severe rocky desertification grasslands (Figure 4C,G). In moderate rocky desertification grasslands, litter showed “enrichment-release-enrichment-release” (Figure 4C), and root showed “enrichment–release” (Figure 4G).

The TK of litter and root in the potential, moderate, and severe rocky desertification grasslands all showed “leaching-enrichment-release”. Among them, the release rate of the TK in litter in the moderate rocky desertification grassland was −2.12%, showing a net enrichment state (Figure 4D,H).

With the deepening of rocky desertification, the return amounts of litter OC and TN in the three types of rocky desertification grasslands showed gradually decreasing trends. The return amounts of TP and TK in the moderate rocky desertification grasslands were the lowest, at only 40% and 12.68% of those in the potential rocky desertification grasslands, respectively. With the deepening of rocky desertification, the return amounts of root OC, TN, TP, and TK showed the same trends, all reaching maximum values in the moderate rocky desertification grasslands with maximum values of 314.64, 5.07, 0.43, and 3.61 g·m^−2^, respectively (Table 3). The OC, TN, TP, and TK released by litter and root decomposition were 18.93–263.03 g·m^−2^·yr^−1^, 1.79–5.59 g·m^−2^·yr^−1^, 0.18–0.47 g·m^−2^·yr^−1^, and 0.66–3.70 g·m^−2^·yr^−1^.

**Figure 5:**
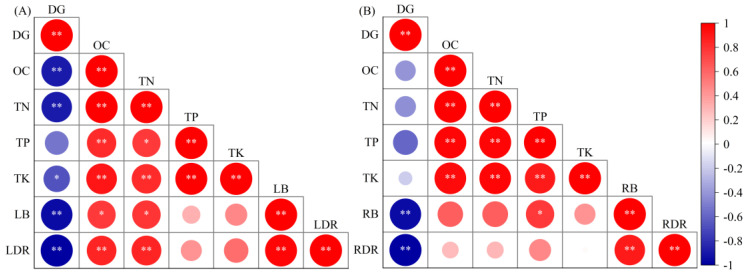
Correlation analysis between the degree of rocky desertification (DG) and litter (**A**) and root (**B**) biomass (litter—LB, root—RB), decomposition rate (litter—LDR, root—RDR), and organic carbon (OC), total nitrogen (TN), total phosphorus (TP), and total potassium (TK) return. Significance level, * *p* < 0.05, ** *p* < 0.01.

The correlation analysis among the various indicators of litter and root (Figure 5) indicated that the degree of rocky desertification showed a significantly negative correlation (*p* < 0.05) with the return amounts of litter OC, TN, and TK, litter biomass, and the annual decomposition rate of litter. Excluding the degree of rocky desertification, the litter OC and TN had a significantly positive correlation (*p* < 0.05) with all the other indicators. There was no significant correlation between the return amount of the TP in litter and litter biomass or the decomposition rate of litter (Figure 5A). The degree of rocky desertification had an insignificantly negative correlation with the return amounts of root nutrients and a significantly negative correlation with root biomass and the annual decomposition rate (*p* < 0.05). There was a significantly positive correlation (*p* < 0.05) among the return amounts of root OC, TN, TP, and TK. Root biomass and the annual decomposition rate had no significant correlation with the return amounts of root OC, TN, TP, and TK (Figure 5B).

## 3. Discussion

### 3.1. Decomposition of Litter and Root in the Different Rocky Desertification Grasslands

The decomposition of litter and root is a key process in ecosystem nutrient cycling that connects soil–plant feedback [19]. This process is comprehensively affected by biological (e.g., soil animals and microorganisms) and abiotic (e.g., climate and soil environment) factors [8,9]. The quality of litter, soil, and microorganisms determines the decomposition rate of litter and root [19]. In this study, in the three types of rocky desertification grasslands, during the initial stage of decomposition (0–120 days), the root mass decreased by 33.99–43.90%, and the litter mass decreased by 23.00–41.41%. During the later stage of decomposition (180–300 days), the root mass decreased by 5.00–9.91%, and the litter mass decreased by 8.42–13.18%. It had obvious stages, with faster decomposition during the early stage and a slower decomposition during the later stage [14]. This was primarily because the content of substances such as carbohydrates in the litter and root was relatively high during the initial stage of decomposition. As the decomposition proceeded, most soluble substances were consumed, and refractory substances such as lignin and cellulose were slowly degraded under the action of microorganisms [20]. In addition, the root decomposition rate was higher than that of litter. The carbon-to-nitrogen ratio is one of the predictable factors that affects litter decomposition. A higher initial nitrogen concentration will make its decomposition rate faster [21]. In this study, the initial nitrogen content of the root was much higher than that of the litter. However, litter decomposition occurs on the soil surface, while the root is in full contact with the soil, and the activities of soil animals and microorganisms are more severe, making its decomposition rate greater than that of the litter.

In this study, the annual litter decomposition rate in the three types of rocky desertification grasslands was 49.79–69.98%, and the annual decomposition rate of root was 64.09–73.64%. The larger the decomposition coefficient, *k*, the faster the decomposition rate; that is, with the deepening of rocky desertification, the decomposition rate of litter and root gradually decreases. With the intensification of rocky desertification, soil and vegetation conditions become fragile, the rock exposure rate increases, the soil layer is thinner, and the number of soil microorganisms decreases. Plants allocate more biomass to their roots [22,23,24]. Therefore, litter production decreases, and the decomposition rate slows down [25]. Studies suggest that because litter is more fragile than roots and has a higher nutrient content, its decomposition rate is much higher than that of root [1,26]. This study obtained the opposite result. The position of litter above and below the ground affected the litter decomposition rate [27]. In this study, the litter was on the soil surface, while the root was 5–10 cm deep in the soil. The soil surface in the rocky desertification area was dry, and the microbial activity was weaker than that in the soil, resulting in a slow decomposition rate. In this study, the root turnover rate in the severe rocky desertification grassland was 0.80 yr^−1^, which was much lower than that in the non-karst ecosystem, which was consistent with the previous research results [28,29]. The low water supply in the karst habitat may limit microbial activity and thereby inhibit litter decomposition [30,31]. The initial nitrogen concentration of the root is another important factor affecting its decomposition rate [21]. The initial nitrogen content of the root in severe desertification grassland is lower than that in potential and moderate desertification grassland, leading to a decrease in turnover rate. There is a significant positive correlation between soil animals and litter decomposition rate [32], and the density of soil animals gradually decreases with the deepening of rocky desertification [33].

### 3.2. Contribution of Litter and Root Decomposition in Different Rocky Desertification Grasslands to Soil Nutrients

Litter nutrient release is an important way to maintain the soil biochemical process in an ecosystem [14]. In different rocky desertification grasslands, due to different physical and chemical processes, the nutrient release dynamics during litter and root decomposition showed different trends. Except for the total potassium in the moderate rocky desertification grassland, the nutrient residual rates of litter and root show decreasing trends [34]. These nutrients are released under the combined action of physical leaching and microbial metabolism [35,36].

The nutrient input of litter depends on factors such as litter productivity, nutrient content, and decomposition rate. In this study, the OC and TN inputs of litter was 38.69–66.35 g·m^−2^·yr^−1^ and 0.47–0.68 g·m^−2^·yr^−1^, respectively, lower than the C and N inputs of litter in forest ecosystems (C: 192.03–249.85 g·m^−2^·yr^−1^, N: 2.10–2.68 g·m^−2^·yr^−1^), and the TP, TK inputs of litter were comparable to the P and K inputs in forest ecosystems [37]. The root OC and TN inputs were 110.24–314.64 g·m^−2^·yr^−1^ and 1.32–5.07 g·m^−2^·yr^−1^, respectively, lower than the root C (360.0 g·m^−2^·yr^−1^) and N (8.1 g·m^−2^·yr^−1^) inputs in forest ecosystems in North China [38]. This is because the root and litter production of rocky desertification grasslands is significantly lower than forest ecosystems. Furthermore, the higher exposed rock rate caused the soil to have shallow layers and special drought. Therefore, production and drought in rocky desertification grasslands could decrease the OC and TN inputs.

In this study, during the first 120 days of decomposition, except for organic carbon, the release rates of N, P, and K in the root were generally higher than those in the litter, indicating that the microorganisms involved in the root decomposition in the soil were more likely to obtain water, organic matter, and mineral nitrogen than the microbial decomposers in the fallen leaves on the soil surface [20,39]. Studies have shown that litter is more inclined to slow nutrient release and has a better nutrient retention mechanism, while the C release rate in roots is slower. This indicates that roots are more conducive to carbon protection in ecosystems [1]. During the first 180 days of litter decomposition, net N mineralization is generally manifested, indicating that there may be a N limitation in moderate and severe rocky desertification grasslands in this study area [39]. Decomposers obtain exogenous nitrogen from the litter and convert it into microbial biomass when nitrogen is scarce, resulting in an increase in nitrogen residue [40]. When N is in excess relative to microbial demand, net nitrogen mineralization increases and nitrogen nutrient release occurs.

The study found that with the deepening of rocky desertification, the contribution of litter to soil nutrients gradually decreased, while the contribution of root to soil nutrients first increased and then decreased. Compared with non-rocky desertification areas, plants allocate more biomass to underground root [22], resulting in a decrease in litter production and a subsequent decrease in its decomposition amount. Sheng et al. pointed out that due to the aggregation effect of exposed rocks in a rocky desertification environment, the soil in a rocky desertification environment shows a process of degradation first and then improvement [41]. When the soil is deficient in nutrients, plants will increase root biomass and turnover rate to improve the absorption capacity of soil nutrients, accelerate root turnover, and promote decomposition.

## 4. Materials and Methods

### 4.1. Overview of the Study Area

**Figure 6:**
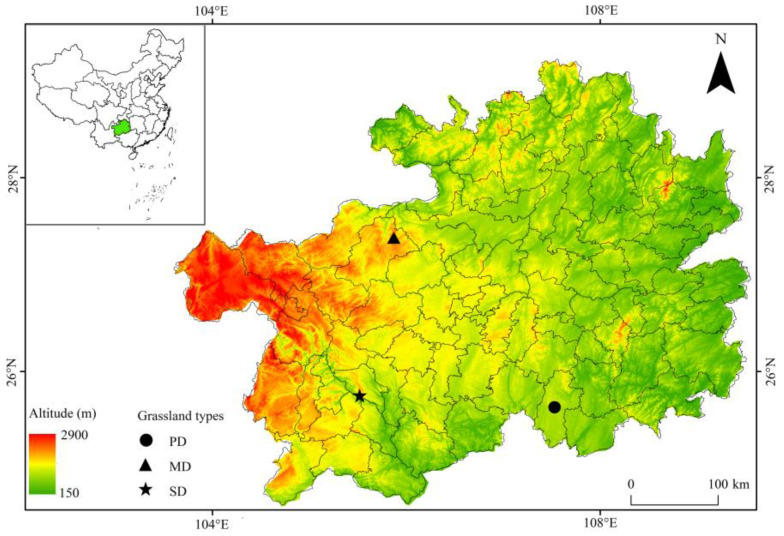
Location and basic information of experiment sites in this study.

**Figure 7:**
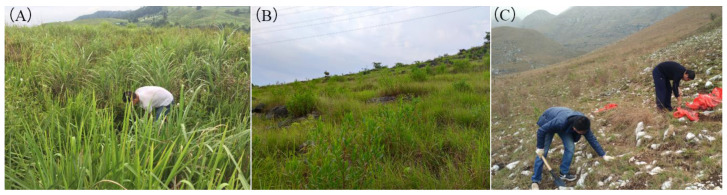
Photos of the potential (**A**), moderate (**B**), and severe (**C**) rocky desertification grasslands.

The study area was located in Guizhou Province in the southwest of China. Carbonates are widely distributed within the region, and the karst landform is highly developed. The terrain is high in the west and low in the east, sloping from the central part toward the north, east, and south. The average altitude is 1100 m. It has a mid-subtropical monsoon humid climate with an average annual temperature of 15 °C and an average annual precipitation of 1133 mm. The soil type is primarily yellow soil with a pH ranging from 4.60 to 6.80. Potential, moderate, and severe rocky desertification grasslands were selected as the research objects (Figure 6). Among these, the potential rocky desertification grassland (PD, Figure 7A) was located in the Grassland Experiment Technology Extension Station in Guizhou Province with geographical coordinates of 107°32′ E, 25°38′ N. The soil pH ranges from 5.42 to 6.07. The constructive species of the grassland is *Miscanthus floridulus*, covering 65% of the grassland, and the associated species is *Cymbopogon caesius*. The moderate rocky desertification grassland (MD, Figure 7B) was located in the Youshahe Tourist Scenic Area, with geographical coordinates of 105°52′ E, 27°23′ N. The pH ranges from 4.61 to 5.32. The constructive species of the grassland is *Miscanthus floridulus*, covering 55% of the grassland, and the associated species are *Osbeckia opipara* and *Juncus effusus*. The severe rocky desertification grassland (SD, Figure 7C) was located in the pasture of Shengshi Animal Husbandry Co., Ltd., An’shun, China, with geographical coordinates of 105°31′ E, 25°45′ N. The pH ranges from 6.2 to 6.8. The constructive species of the grassland is *Cymbopogon caesius*, covering 40% of the grassland, and the associated species are *Magnolia multiflora* and *Dodonaea viscosa*.

### 4.2. Experimental Design

#### 4.2.1. Litter and Root Biomass

In January 2017, three 25 m × 25 m sample plots were established in each of the three grasslands. Three 1 m × 1 m quadrats were set along the diagonal of each sample plot. The litter in the quadrats was collected, which was the litter biomass. In addition, a 50 cm × 50 cm sampling point was established in each quadrat after the litter was collected. The continuous soil column method was used, and the soil layer within the sampling point was excavated every 10 cm in sequence until there were few roots. The collected soil samples were placed on a fine sieve (mesh size 1.0 mm) and rinsed with water to leave only the clean root. The root sample was separated into living and dead root using visual criteria, such as texture, elasticity, and color. Living root was resilient, flexible, fleshy, and shiny. A root was classified dead if it was limp or crumbled easily [42]. After drying at 80 °C to a constant weight, they were weighed. Based on the biomass of dead root and living root obtained from each sampling, the annual root mortality and annual root production were determined.

The litter production was determined using the collector method. In January 2017, five 1 m × 1 m litter collectors (mesh size 0.1 mm) were placed in each 25 m × 25 m sample plot mentioned above. The collectors were 2 cm away from the ground surface and were collected once every two months for a total of six times. They were brought back to the laboratory, and impurities, such as stones and insects in the litter, were picked out. They were placed in an oven, dried at 80 °C for 48 h, and weighed to calculate the annual litter production.

#### 4.2.2. Soil Sampling

In addition, three sampling points were selected along the diagonal in the above quadrats. The litter on the soil surface was removed. Using the soil auger method, approximately 300 g of the soil samples from the 0–10 cm soil layer was drilled. The collected soil samples were air-dried in a cool place, and impurities, such as stones and root, were picked out and sieved through 0.15 mm for an analysis of the contents of the soil organic carbon (SOC), total nitrogen (TN), total phosphorus (TP), and total potassium (TK).

#### 4.2.3. Litter and Root Decomposition

The nylon mesh bag method was used for the litter and root decomposition experiment. In March 2017, 5.0 g of dried litter and 3.0 g of dried root were accurately weighed and placed in nylon mesh bags with a specification of 10 cm × 10 cm and a pore size of 0.2 mm. In each sample plot, three locations along the diagonal were established for the litter and root decomposition bags, and there were 30 litter and root decomposition bags at each location. The litter decomposition bags were fixed on the soil surface, and the root decomposition bags were fixed at a depth of 10–15 cm. Every two months, five litter decomposition bags and five root decomposition bags were retrieved from each location. For the retrieved decomposition bags, impurities were removed, transferred to envelopes, dried at 65 °C to a constant weight, crushed, and passed through a 200-mesh sieve, and the contents of organic carbon (OC), TN, TP, and TK in the litter were determined.

### 4.3. Laboratory Analysis

The OC in the soil litter and root was determined using the external heating method with K_2_Cr_2_O_7_. The TN was determined using the Kjeldahl nitrogen determination method. The TP was determined using the molybdenum-antimony anti-colorimetric method. The TK was determined using the flame photometer method.

### 4.4. Data Processing and Analysis

The calculation equation for the decomposition rate of litter and root is

$$R=1-W_{t}/W_{0}\times 100\%$$

where *R* is the decomposition rate of litter and root; *W*_0_ is the initial dry weight of litter and root (g); and *W_t_* is the dry weight of litter and root at time *t* (g).

The residual amount of litter and root was fitted using the improved Olson negative exponential model

$$W_{t}=ae^{-kt}$$

where *W_t_* is the residual amount of litter and root; *a* is the fitting parameter; *k* is the decomposition coefficient; and *t* is the time.

The calculation equations for the turnover rate of litter and root are

$$T_{l}=L/S\;M=M_{max}-M_{min}+D\;P=P_{max}-P_{min}+M\;T_{r}=P/Y$$

where *T_l_* is the turnover rate of litter; *L* is the biomass of litter (current standing stock); *S* is the annual production of litter; *T_r_* is the turnover rate of root; *M* is the annual mortality of root; *M_max_* is the maximum value of the current standing stock of dead root; *M_min_* is the minimum value of the current standing stock of dead root; *D* is the annual decomposition amount of litter; *P* is the root production; and *P_max_*, *P_min_*, and *Y* are the maximum, minimum, and average values of the current standing stock of living roots, respectively.

The calculation equation for the nutrient residual rate of litter and root is

$$N=(M_{t}\times C_{t})/(M_{0}\times C_{0})\times 100\%,$$

where *N* is the nutrient residual rate of litter and roots; *M_t_* is the dry weight of litter and roots at time *t* (g); *C_t_* is the nutrient content of litter and roots at time *t* (%); *M*_0_ is the initial dry weight of litter and root (g); and *C*_0_ is the initial nutrient content of litter and root (%).

The calculation equations for the nutrient return amount of litter and roots are

$$C_{l}=D\times C_{0}\times T_{l}\;C_{r}=\left(M\times C_{0}-R\times C_{t}\right)\times T_{r}\;R=M-D$$

where *C_l_* is the nutrient return amount of litter (g·m^−2^); *C_r_* is the nutrient return amount of root; *D* is the annual decomposition amount of litter (g·m^−2^·yr^−1^); *C*_0_ is the initial nutrient content of litter or root (%); *R* is the biomass of undegraded root (g·m^−2^·yr^−1^); *C_t_* is the content of undegraded root (%); *M_max_* is the maximum value of the current standing stock of dead root; and *M_min_* is the minimum value of the current standing stock of dead root.

A statistical analysis was performed using SPSS 26.0 software. A one-way analysis of variance (ANOVA) and multiple comparisons (ANOVA–Duncan) method were used to test the differences in the soil nutrients, litter production, root biomass, mortality, decomposition residual amount, decomposition rate, nutrient content, and return amount of different rocky desertification grasslands. The significance level was *p* = 0.05. The Pearson method was used to detect the correlation between the degree of rocky desertification and litter production, the root biomass, the decomposition rate, and the nutrient return amount. Exponential curve fitting was used to analyze the decomposition process of litter and root. The Origin 2022 software was used for mapping. All data in the charts are expressed as means ± standard deviation (Mean ± SD).

## 5. Conclusions

This study found that the decomposition rates of litter and root gradually decreased with the decomposition time, and the degree of rocky desertification was negatively correlated with the biomass, decomposition rate, and nutrient return amount of litter and root. After 120 days of decomposition, the nutrient preservation mechanism of OC, TN, TP, and TK in the root was greater than that of the litter in the severe rocky desertification grassland. The nutrient preservation mechanisms of TN, TP, and TK were greater in root than in litter in moderate rocky desertification grassland. The decomposition rate of litter and root was greater than 50% after 300 days and the release processes of OC, TN, TP, and TK in the litter and root varied greatly. With the exception of TK in the litter of moderate rocky desertification areas, they all showed a release state. The contribution of root to soil nutrients was greater than that of litter. The contribution of litter and root decomposition to soil nutrients followed the trend of moderate rocky desertification grassland > potential rocky desertification grassland > severe rocky desertification grassland. The OC, TN, TP, and TK released by litter and root decomposition were 18.93–263.03 g·m^−2^·yr^−1^, 1.79–5.59 g·m^−2^·yr^−1^, 0.18–0.47 g·m^−2^·yr^−1^, and 0.66–3.70 g·m^−2^·yr^−1^. This study promotes our understanding of the decomposition of litter and root in rocky desertification grasslands and provides valuable data for the protection of these areas.

## Figures and Tables

**Table 1:** Litter and root productivity, annual decomposition amount, and turnover rate in potential (PD), moderate (MD), and severe (SD) rocky desertification grasslands. Different lowercase letters indicate significant differences in different rocky desertification grasslands.

	Grassland Type	Productivity (g·m^−2^·yr^−1^)	Decomposition Amount (g·yr^−1^)	Turnover Rate (yr^−1^)
Litter	PD	222 ± 6.14 a	454 ± 25.7 a	0.50 ± 0.01 b
	MD	186 ± 3.85 b	318 ± 27.8 b	0.47 ± 0.02 b
	SD	157 ± 7.27 c	269 ± 21.6 b	0.57 ± 0.05 a
Root	PD	4386 ± 532 a	294 ± 59.3 b	1.51 ± 0.18 a
	MD	2985 ± 251 b	549 ± 20.5 a	1.39 ± 0.07 a
	SD	1167 ± 141 c	273 ± 32.6 b	0.80 ± 0.08 b

**Table 2:** Regression decomposition model of litter in potential rocky desertification (PD), moderate rocky desertification (MD), and severe rocky desertification (SD) grasslands.

	Grassland Types	Fitted Equation	*R* ^2^	50% Decomposition Time (yr)	95% Decomposition Time (yr)	Annual Decomposition Rate (%)
	PD	*y* = 4.64*e*^−1.13*t*^	0.90	0.55	2.59	69.98
Litter	MD	*y* = 4.64*e*^−0.90*t*^	0.88	0.69	3.26	62.14
	SD	*y* = 4.89*e*^−0.67*t*^	0.98	1.01	4.46	49.79
	PD	*y* = 2.77*e*^−1.25*t*^	0.89	0.49	2.33	73.64
Root	MD	*y* = 2.69*e*^−1.02*t*^	0.78	0.57	2.84	67.61
	SD	*y* = 2.89*e*^−0.99*t*^	0.96	0.66	3.00	64.09

**Table 3:** Annual returns of organic carbon (OC), total nitrogen (TN), total phosphorus (TP), and total potassium (TK) of litter and root in potential (PD), moderate (MD), and severe (SD) rocky desertification grasslands. Different lowercase letters indicate significant differences in different rocky desertification grasslands.

	Grassland Types	OC (g·m^−2^)	TN (g·m^−2^)	TP (g·m^−2^)	TK (g·m^−2^)
Litter	PD	66.35 ± 3.75 a	0.68 ± 0.04 a	0.10 ± 0.01 a	0.71 ± 0.04 a
	MD	42.45 ± 3.71 b	0.52 ± 0.05 b	0.04 ± 0.00 c	0.09 ± 0.01 c
	SD	38.69 ± 3.11 b	0.47 ± 0.04 b	0.07 ± 0.01 b	0.28 ± 0.02 b
Root	PD	196.67 ± 39.58 b	2.94 ± 0.59 b	0.31 ± 0.06 b	1.02 ± 0.2 b
	MD	314.64 ± 11.75 a	5.07 ± 0.19 a	0.43 ± 0.02 a	3.61 ± 0.13 a
	SD	110.24 ± 13.13 c	1.32 ± 0.16 c	0.11 ± 0.01 c	0.38 ± 0.04 c

## Data Availability

The datasets generated and analyzed during the current study are available from the corresponding author on reasonable request.

## References

[1] Sun T., Hobbie S.E., Berg B., Zhang H., Httenschwiler S. (2018). Contrasting dynamics and trait controls in first-order root compared with leaf litter decomposition. Proc. Natl. Acad. Sci. USA.

[2] Guo L.L., Deng M.F., Yang S., Liu W.X., Wang X., Wang J., Liu L.L. (2021). The coordination between leaf and fine root litter decomposition and the difference in their controlling factors. Glob. Ecol. Biogeogr..

[3] Loranger G., Ponge J.F., Imbert D., Lavelle P. (2002). Leaf decomposition in two semi-evergreen tropical forests: Influence of litter quality. Biol. Fertil..

[4] Gessner M.O., Swan C.M., Dang C.K., Mckie B.G., Bardgett R.D., Wall D.H., Hättenschwiler S. (2010). Diversity meets decomposition. Trends Ecol. Evol..

[5] Chapin F.S., Matson P.A., Vitousek P.M. (2012). Principles of Terrestrial Ecosystem Ecology.

[6] Vogt K.A., Grier C.C., Vogt D.J. (1986). Production, turnover, and nutrient dynamics of above-and belowground detritus of world forests. Adv. Ecol. Res..

[7] Ruess R.W., Cleve K.V., Yarie J., Viereck L.A. (1996). Contributions of fine root production and turnover to the carbon and nitrogen cycling in taiga forests of the Alaskan interior. Can. J. For. Res..

[8] Hyvönen R., Olsson B.A., Lundkvist H., Staaf H. (2000). Decomposition and nutrient release from *Picea abies* (L.) Karst. and *Pinus sylvestris* L. logging residues. For. Ecol. Manag..

[9] Penner J.F., Frank D.A. (2019). Litter decomposition in Yellowstone grasslands: The roles of large herbivores, litter quality, and climate. Ecosystems.

[10] Jiang Z.C., Lian Y.Q., Qin X.Q. (2014). Rocky desertification in Southwest China: Impacts, causes, and restoration. Earth Sci. Rev..

[11] Gao J.F., Su X.L., Xiong K.L. (2011). Grasslands eco-environment and stockbreeding development in the karst areas of Guizhou province. Acta Pratacul. Sin..

[12] Hättenschwiler S., Tiunov A.V., Scheu S. (2005). Biodiversity and litter decomposition in terrestrial ecosystems. Annu. Rev. Ecol. Evol. Syst..

[13] Zhu X.A., Zou X., Lu E.F., Deng Y., Luo Y., Chen H., Liu W.J. (2021). Litterfall biomass and nutrient cycling in karst and nearby non-karst forests in tropical China: A 10-year comparison. Sci. Total Environ..

[14] Pang Y., Tian J., Lv X.Y., Wang R., Wang D.X., Zhang F.F. (2022). Contrasting dynamics and factor controls in leaf compared with different-diameter fine root litter decomposition in secondary forests in the Qinling Mountains after 5 years of whole-tree harvesting. Sci. Total Environ..

[15] Lv C., Saba T., Wang J.Y., Hui W.K., Kang X.K., Xie Y.X., Wang H.L., Gong W. (2022). Conversion effects of farmland to Zanthoxylum bungeanum plantations on soil organic carbon fractions in the arid valley of the upper reaches of the yangtze river, China. Catena.

[16] Contos P., Murphy N.P., Kayll Z.J., Morgan T., Vido J.J., Decker O., Gibb H. (2024). Rewilding soil and litter invertebrates and fungi increases decomposition rates and alters detritivore communities. Ecol. Evol..

[17] Liu Y.L., Wang K.B., Dong L.B., Li J.W., Wang X.Z., Shangguan Z.P., Qu B.D., Deng L. (2023). Dynamics of litter decomposition rate and soil organic carbon sequestration following vegetation succession on the Loess Plateau, China. Catena.

[18] Glass N., Oliveira E.D.D., Molano-Flores B., Matamala R., Whelan C.J., Gonzalez-Meler M.A. (2023). Root litter decomposition rates and impacts of drought are regulated by ecosystem legacy. Appl. Soil Ecol..

[19] Fu Y.M., Feng F.J., Zhang X.Y., Qi D.D. (2021). Changes in fine root decomposition of primary *Pinus koraiensis* forest after clear cutting and restoration succession into secondary broad-leaved forest. Appl. Soil Ecol..

[20] Silver W.L., Miya R.K. (2001). Global patterns in root decomposition: Comparisons of climate and litter quality effects. Oecologia.

[21] Mun S., Lee E.J. (2020). Litter decomposition rate and nutrient dynamics of giant ragweed (*Ambrosia trifida* L.) in the non-native habitat of South Korea. Plant Soil.

[22] Ni J., Luo D.H., Xia J., Zhang Z.H., Hu G. (2015). Vegetation in karst terrain of southwestern China allocates more biomass to roots. Solid Earth.

[23] Zhu X.A., Shen Y.X., He B.B., Zhao Z.M. (2017). Humus soil as a critical driver of flora conversion on karst rock outcrops. Sci. Rep..

[24] Liu J., Shen Y.X., Zhu X.A., Zhao G.J., Zhao Z.M., Li Z.J. (2019). Spatial distribution patterns of rock fragments and their underlying mechanism of migration on steep hillslopes in a karst region of Yunnan Province, China. Environ. Sci. Pollut. Res..

[25] Song X.W., Gao Y., Wen X.F., Guo D.L., Yu G.R., He N.P., Zhang J.Z. (2017). Carbon sequestration potential and its eco-service function in the karst area, China. J. Geogr. Sci..

[26] Wang H., Liu S., Mo J. (2010). Correlation between leaf litter and fine root decomposition among subtropical tree species. Plant Soil.

[27] Berenstecher P., Araujo P.I., Austin A.T. (2021). Worlds apart: Location above-or below-ground determines plant litter decomposition in a semi-arid Patagonian steppe. J. Ecol..

[28] Yuan Z.Y., Chen H.Y. (2010). Fine root biomass, production, turnover rates, and nutrient contents in boreal forest ecosystems in relation to species, climate, fertility, and stand age: Literature review and meta-analyses. Crit. Rev. Plant Sci..

[29] Xiong Y.M., Liu X., Guan W., Liao B.W., Chen Y.J., Li M., Zhong C.R. (2017). Fine root functional group based estimates of fine root production and turnover rate in natural mangrove forests. Plant Soil.

[30] Guo Y.L., Chen H.Y.H., Mallik A.U., Wang B., Li D.X., Xiang W.S., Li X.K. (2019). Predominance of abiotic drivers in the relationship between species diversity and litterfall production in a tropical karst seasonal rainforest. For. Ecol. Manag..

[31] Yan Y.J., Dai Q.H., Hu G., Jiao Q., Mei L.N., Fu W.B. (2020). Effects of vegetation type on the microbial characteristics of the fissure soil-plant systems in karst rocky desertification regions of SW China. Sci. Total Environ..

[32] Xin W.D., Yin X.Q., Song B. (2012). Contribution of soil fauna to litter decomposition in Songnen sandy lands in northeastern China. J. Arid. Environ..

[33] Dai X.Y., Tang J., Song L.H. (2019). A review on ecological studies of soil fauna in karst region, Southwest China. Chin. J. Ecol..

[34] Bravo-Oviedo A., Ruiz-Peinado R., Onrubia R., del Río M. (2017). Thinning alters the early-decomposition rate and nutrient immobilization-release pattern of foliar litter in Mediterranean oak-pine mixed stands. For. Ecol. Manag..

[35] Fujii S., Makita N., Mori A.S., Takeda H. (2016). A stronger coordination of litter decomposability between leaves and fine roots for woody species in a warmer region. Trees.

[36] Zhuang L.Y., Yang W.Q., Wu F.Z., Tan B., Zhang L., Yang K.J., He R.Y., Li Z.J., Xu Z.F. (2018). Diameter-related variations in root decomposition of three common subalpine tree species in southwestern China. Geoderma.

[37] Alvafritz L., Hertel D. (2024). Impacts of land use history on leaf litter input, chemical composition, decomposition and related nutrient cycling in young and old secondary tropical lowland rainforests (Sumatra, Indonesia). Plant Soil.

[38] Guo L.B., Wang M.B., Gifford R.M. (2007). The change of soil carbon stocks and fine root dynamics after land use change from a native pasture to a pine plantation. Plant Soil.

[39] Lin C.F., Yang Y.S., Guo J.F., Chen G.S., Xie J.S. (2011). Fine root decomposition of evergreen broadleaved and coniferous tree species in mid-subtropical China: Dynamics of dry mass, nutrient and organic fractions. Plant Soil.

[40] Schleuss P.M., Widdig M., Biederman L.A., Borer E.T., Crawley M.J., Kirkman K.P., Seabloom E.W., Wragg P.D., Spohn M. (2021). Microbial substrate stoichiometry governs nutrient effects on nitrogen cycling in grassland soils. Soil Biol. Biochem..

[41] Sheng M.Y., Liu Y., Xiong K.N. (2013). Response of soil physical and chemical properties to karst rocky desertification succession in southern South China Karst. Acta Ecol. Sin..

[42] Persson H.A., Stadenberg I. (2010). Fine root dynamics in a Norway spruce forest (*Picea abies* (L.) Karst) in eastern Sweden. Plant Soil.

